# Retraction Note: NOTCH localizes to mitochondria through the TBC1D15-FIS1 interaction and is stabilized via blockade of E3 ligase and CDK8 recruitment to reprogram tumor-initiating cells

**DOI:** 10.1038/s12276-026-01834-9

**Published:** 2026-08-18

**Authors:** Hye Yeon Choi, Yicheng Zhu, Xuyao Zhao, Simran Mehta, Juan Carlos Hernandez, Jae-Jin Lee, Yi Kou, Risa Machida, Mauro Giacca, Giannino Del Sal, Ratna Ray, Hyungjin Eoh, Stanley M. Tahara, Lin Chen, Hidekazu Tsukamoto, Keigo Machida

**Affiliations:** 1 Departments of Molecular Microbiology and Immunology, University of Southern California, Los Angeles, CA USA; 2 Viterbi School of Engineering, University of Southern California, Los Angeles, CA USA; 3 International Centre for Genetic Engineering and Biotechnology, Trieste, Italy; 4 Department of Life Sciences, University of Trieste, 34127 Trieste, Italy; 5 International Centre for Genetic Engineering and Biotechnology (ICGEB), Area Science Park-Padriciano, Trieste, Italy; 6 IFOM ETS, The AIRC Institute of Molecular Oncology, Milan, Italy; 7 Saint Louis University, School of Medicine, St Louis, MO USA; 8 Department of Pathology, University of Southern California, Los Angeles, CA USA; 9 Southern California Research Center for ALPD and Cirrhosis, Los Angeles, CA USA

Retraction Note to: *Experimental & Molecular Medicine* https://doi.org/10.1038/s12276-024-01174-6, published online 27 February 2024

The Editor-in-Chief has retracted this article because of concerns regarding the figures presented in this work. These concerns call into question the article’s overall scientific soundness. An investigation conducted after its publication discovered the following issues:

- the NICD band in Figure 6l appears partially to overlap, after a vertical flip, with the PTEN band in Fig. 5B in ref. ^1^;
- the Actin band in Fig. 6l appears to overlap with the aPKCζ band in Fig. 5e in ref. ^2^;
- the second mouse in the 10 mg/kg row of Fig. 6i appears to be identical to the third mouse in the sh-Ezh2 row of Fig. 4D in ref. ^3^;
- the third mouse in the 20 mg/kg row of Fig. 6i appears to be identical to the mouse shown in the E2f1 shRNA panel in Fig. 3G in ref. ^4^.

The Editor-in-Chief therefore no longer has confidence in the integrity of the research presented in this article.

Keigo Machida has stated on behalf of all the authors that they agree with this retraction.
